# History of the development of radiotherapy in Latin America

**DOI:** 10.3332/ecancer.2017.784

**Published:** 2017-11-30

**Authors:** Luis Pinillos, Joseph A Pinto, Gustavo Sarria

**Affiliations:** 1 Departamento de Radioterapia, Oncosalud-AUNA, Av Paseo de la República 3650, San Isidro, Lima 15046, Peru; 2 Unidad de Investigación Básica y Traslacional, Oncosalud-AUNA, Av Guardia Civil 571, San Borja, Lima 15036, Peru

**Keywords:** radiation oncology, Latin American, cancer care, history

## Abstract

Radiotherapy was the first nonsurgical treatment for malignant tumours and represents one of the oldest disciplines of oncology. In Latin America, as in many parts of the world, the history of modern oncology begins with the implementation of radiation therapy facilities. The development of radiotherapy in Latin America was possible thanks to the seminal work of radiation oncologists in different countries. As a large territory, there is a need to implement modern facilities and equipment, but unfortunately there are disparities in the access and quality of radiotherapy services across Latin America and even within individual countries. In this review, we describe the history, challenges and success in the implementation of radiotherapy and the frustration caused by the lack of facilities in several Latin American countries.

## Background

Soon after the discoveries of x-rays and radium, biological effects were detected and many diseases, including cancer, were subjected to radiation exposure with the intent to cure [7]. Radiotherapy was developed as a trial and error experience, and it was born as a scientific discipline during the International Congress of Oncology in Paris (1922), where its scientific basis (like fractionation) was presented [9]. Since then, radiotherapy has developed with increasing precision of dosimetry, knowledge of biological effects, increasing energy of x-rays, technological development of machines, imaging improvements and computational capacity. It is now a fundamental component of oncological treatment, and thanks to advances in precision radiation delivery, it is now possible to administer higher doses with lower toxicities to healthy tissue, achieving better outcomes [17].

Latin America is a large region located south of Rio Grande, with an area of 22.5 million km^2^. It is estimated that 97% of the population speaks Spanish or Portuguese. One-third of the population is concentrated in large cities, but two-thirds live in small cities and rural areas, with high levels of poverty, insufficient education, fragmented health infrastructures, restricted healthcare coverage (less than 50% with coverage), limited healthcare workers and few cancer registries [15, 24]. The number of cancer deaths between 2012 and 2030 will rise by 67%, more than what is expected in North America (41%) [28]. The ≈620 radiotherapy centres in Latin America are largely insufficient for the more than 600 million inhabitants [27].

In a survey to discover the situation of radiotherapy in the region in 1989, social–economic disparities in the distribution of facilities and equipment were evident, with a very small number of large and sophisticated radiation therapy centres in a few developed geographical areas in comparison to a high number of small centres in less developed regions [25].

In 2007, during the first Congress of ALATRO (Ibero-Latin American Association of Radiation Therapy), the results of a multinational evaluation of radiotherapy in Latin America were presented. The results indicated that quality varies between countries and within countries, much of the devices were technological products of the 1960s, many countries had Cobalt-60 units with low output (< 50 cGy per minute) and there was a lack of therapeutic protocols and a lack of quality assurance accompanied by the inadequate geographic distribution of radiotherapy facilities [20].

In this review article, we will present, as examples, the historical development of radiation oncology, and information regarding professional facilities and equipment in selected Latin American (LATAM) countries.

## History of radiotherapy development in LATAM countries

The development of radiotherapy in Latin America was made possible thanks to governmental and private initiatives. The density of radiotherapy machines by population has been low, but it is increasing in all LATAM countries; however, there is a trend to centralise radiotherapy services to great urban populations. Despite the need for radiation oncologists, many professionals of high quality have emigrated to work in the United States and other developed countries, for example Dr Carlos Perez (past President of ASTRO) and Dr Alvaro-Martinez (past President of the American Brachytherapy Society), both from Colombia.

**Figure 1.**
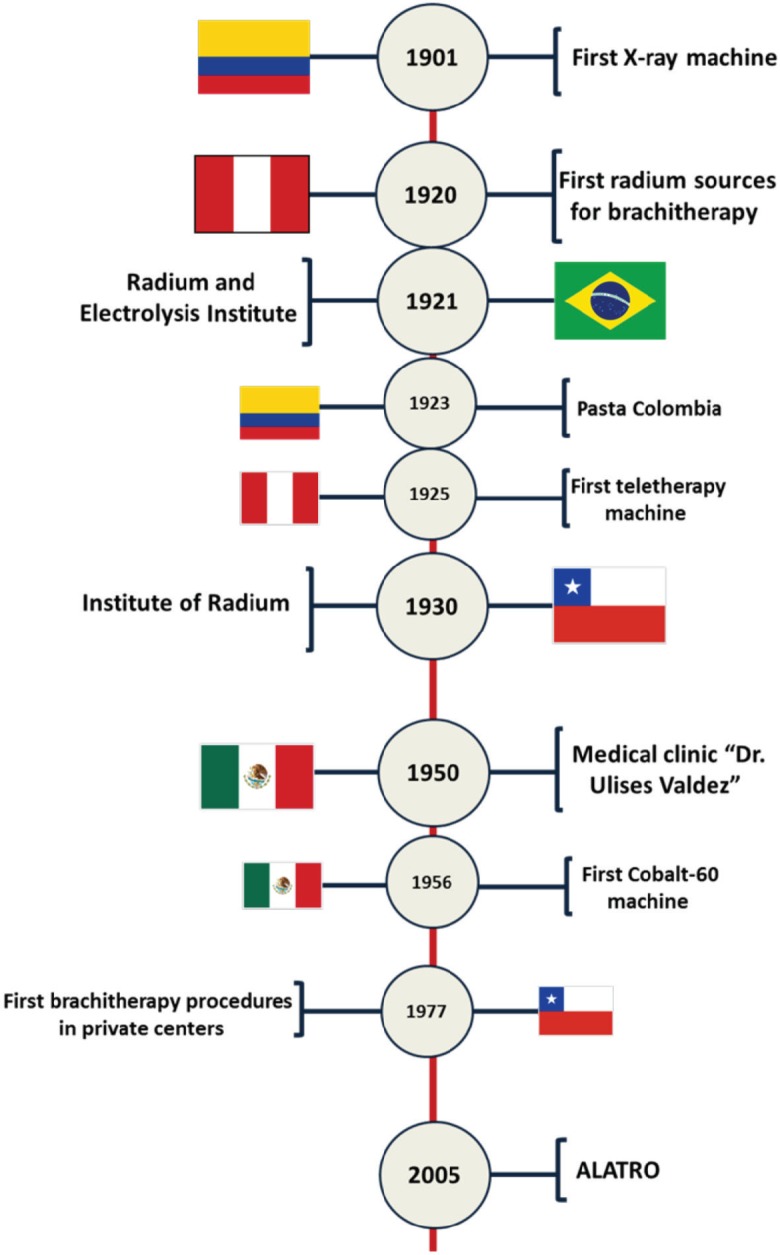
Key events in the development of radiotherapy in Latin America.

We will describe the early implementation of radiotherapy facilities in some LATAM countries (Figure 1)**,** while an updated list of the centres and machines, provided by ALATRO, is given in Table 1. The history of radiotherapy in Uruguay has been recently described in [2].

### Brazil

In 1914, the Radium and Electrolysis Institute was founded by Prof Eduardo Rabello. In 1918, the first high kilovoltage radiotherapy was given by Dr Arnold Campbell. In 1923, Dr Armando Aguinaga began using gynecological radiotherapy. In 1921, the Arnaldo Vieira de Carvalho Institute was founded in Sao Paulo, and in 1930 the Institute acquired a 180-kV machine. In addition, in 1921 the Radium Institute was founded in Minas Gerais, and in 1934, treatments with kilovoltage radiotherapy began. In 1939, the Institute of Medicine and Surgery installed the Service of Roentgen therapy in Parana and Curitiba. In 1924, the Radium and Radiology Institute was inaugurated in Per nambuco, in 1952 the Cancer Hospital was founded, and in 1958 Telecobalt-therapy was offered in the Hospital das Clinicas of the Federal University of Pernambuco. In 1929, the Service of Radiotherapy of the Gaffré e Guinle Hospital was introduced. In 1940, the Cancer and Radiotherapy Institute, A. C. Camargo, was founded by Prof Antonio Prudente. In 1954, the first Telecobalt-therapy was given by Dr Ozolando Judice-Machado. In 1959, the first cobalt-60 machine in the National Cancer Institute was installed, and in 1972 the first linear accelerator was installed at the Oswaldo Cruz Hospital. Currently, Brazilian radiotherapy is one of the most advanced in Latin America, offering high-energy proton radiotherapy, with more than 300 linear accelerators machines, more than 60 cobalt machines and more than 100 HDR brachytherapy machines, distributed all over the country and with the major scientific production in this field in Latin America [16].

### Chile

**Figure 2.**
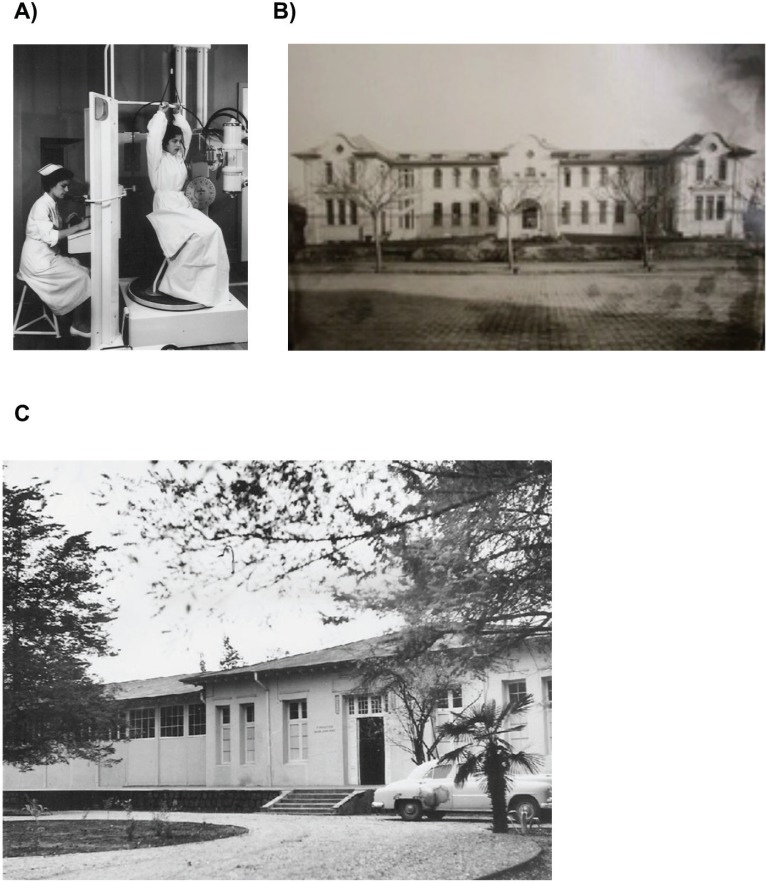
Radiotherapy in Chile. Orthovoltage radiation therapy was one of the first treatments in cancer (a) given at the Radium Institute (b). Lopez Perez Foundation in 1953 (c). Photos courtesy of Dr Juan Solé.

In 1930, the Institute of Radium was inaugurated (Figure 2). In 1956, the first cobalt-60 machine was implemented, and up to 1969, three public hospitals and two private centres obtained cobalt-60 machines. Some private centres initiated offering brachytherapy since 1977. In 1981, the first linear accelerator was implemented (6 MV) in INDISA clinic, and in 1980, it was implemented in a public hospital (Van Buren Hospital). Since 1994, dual linear accelerators were implemented at the Catholic University and in private centres. In 1997, medium dose rate of brachytherapy was given at Hospital Valdivia. Since the year 2000, several IMRT–IGRT machines have been implemented, and in 2007, brachytherapy of high dose was given at the Instituto Nacional del Cancer. In 2010, high-dose rate brachytherapy and Gamma knife was implemented in private centres. One of the best characteristics was the decentralised distribution of radiotherapy facilities; there are 23 radiotherapy facilities with 40 linear accelerators and nine brachytherapy machines, among other RT machines.

### Colombia

**Figure 3.**
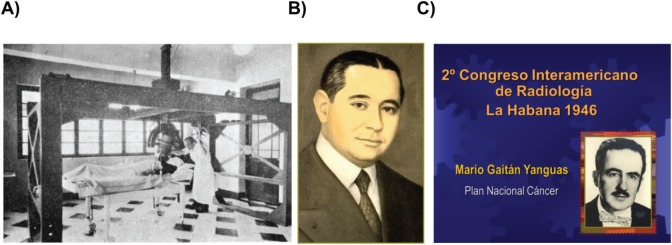
Radiotherapy in Colombia. Telecurietherapy machine at the National Institute of Radium (a). Dr Alfonso Esquerra-Gómez was one of the most important pioneers in radiation oncology in Colombia and Latin America. He developed the ‘Pasta Colombia’ used worldwide (b). Dr Mario Gaitán, Director of the National cancer Institute and Minister of Health, proposed one of the first cancer control plans in 1946 (c). Photos courtesy of Dr Armando Gaitán.

Radiotherapy in Colombia, as a discipline, was influenced by the French and American schools. In 1901, the first x-ray machine was implemented. In 1916, the treatment of cervical cancer with radium was given by Dr Alfonso Esguerra-Gómez. In 1919, skin cancer was treated for the first time with radium by Dr Ricardo Valencia. In 1920, the Service of Radiotherapy of San Juan de Dios Hospital was instated. In 1923, the ‘Pasta Colombia’ (Colombian paste, a mix of paraffin, beeswax and sawdust, which makes a flexible substance) was developed by Dr Alfonso Esguera-Gómez [5]. In 1934, the Radium National Institute was inaugurated with 160 beds, 4 machines of 200 kV, 2 machines of 180 kV and one superficial and one telecurietherapy machine. In 1960, the first cobalt-60 machine was implemented. Dr Mario Gaitan (NCI-Colombia Director and minister of health) presented in Cuba (in 1950) a comprehensive cancer control plan. Currently, Colombia has 55 decentralised centres of radiotherapy with 53 linear accelerators, 22 machines with IMRT capacity and 35 HDR brachytherapy machines, among others [1] (Figure 3).

### Mexico

The first medical centre to provide radio oncological care was the medical clinic of ‘Dr Ulises Valdez’ (1950). Due to the increasing demand for cancer care, the Instituto Nacional de Cancerología was created in 1946. In 1950, Dr Conrado Zuckerman founded the Department of Radiotherapy and Brachytherapy for patients with cervical carcinoma. Some practitioners were working as radiotherapists and surgeons at the same time. In 1956, Dr José Noriega-Limón founded the first cobalt-60 machine [18]. In 1972, in the National University of Mexico, the master programme in physics of radiations and radiologic safety was instated. Up to 1979, 50 centres of radiotherapy with cobalt and cesium machines operated in Mexico. In 1980, the new facility of the National Institute of Cancerology had cobalt-60 machines, linear accelerators, tomography and nuclear medicine. In 1982, in an effort towards cancer care decentralisation, the Onological Centre of the state of Guerrero, sister institution of the National Institute of Cancerology, was inaugurated. In 1993, 30 students graduated from the master programme in medical physics in the Universidad Nacional de México. According to ALATRO, currently, there are 23 public centres offering radiotherapy, with around 30 brachytherapy machines of low and high dose. The data from private centres are not considered [1].

### Peru

Radiotherapy began in 1920 with brachytherapy when Dr Gare (from France) brought to Peru the first radium sources that were then used by Dr Guillermo Gastañeta, Dr Constantino Carvallo and then by Dr Luis Pinillos-Ganoza. Upon inauguration in 1939 of the Instituto Nacional de Enfermedades Neoplasicas (INEN), more radium sources were acquired.

In gynecological treatments, the radium sources were reinserted daily after washing and application of the ‘Bulgarian bacilli’ to prevent infections.

**Figure 4.**
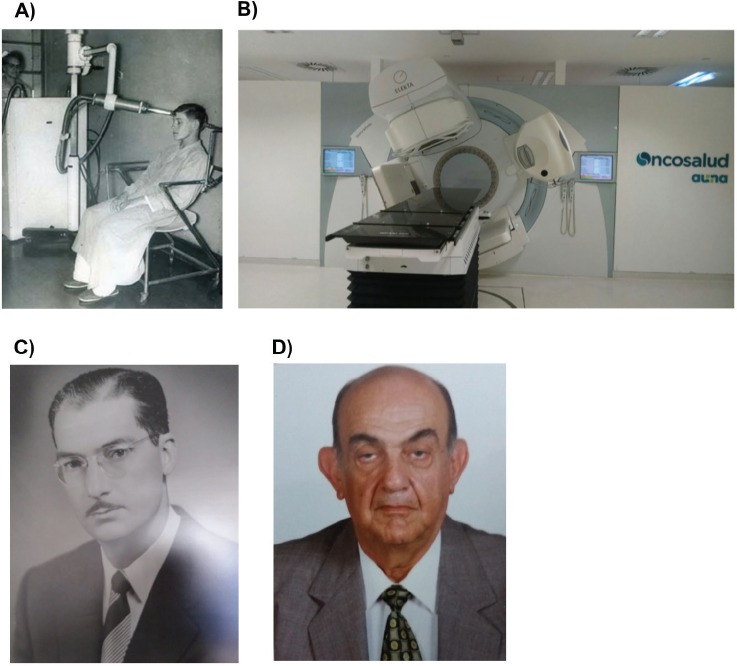
Radiotherapy in Peru. (a) Superficial radiation therapy was given early at INEN. (b) Modern linear accelerator machine at Oncosalud facility. (c) Dr Luis Pinillos-Ganoza was one of the pioneers of the Peruvian radiotherapy. (d) Dr Mayer Zaharia conducted radiotherapy research in several malignancies in Peru.

In the year 1925, the first teletherapy machine (SIEMENS 180 kV) was implemented at the Arzobispo Loayza Hospital. In 1928 and 1933, two similar machines were implemented in private clinics. In 1939, The National Cancer Institute, later renamed the National Institute of Radiotherapy and now named INEN , was inaugurated with 4-kV radiotherapy machines (SIEMENS 200 kV) and one superficial (CHAOUL) (Figure 4). In 1951, the first cobalt-treatment in the world was given and soon after, in 1958, the first cobalt machine was instated at the Radiotherapy Department of INEN.

In 1992, Peru had seven conventional radiotherapy machines and ten megavoltage machines (in a population of ≈22 million inhabitants). In 2003, there were 17 megavoltage machines for 27 million inhabitants [26] and, currently, the Peruvian population is estimated at 31 million inhabitants and counts with 24 megavoltage machines and plans for implementing ten more machines in the near future [1]. Since 2014, intraoperative radiation therapy has been performed at INEN and in Oncosalud, a private group for cancer care and modern treatments like IMRT, IGRT, VMAT, HDR and cobalt are available at three centres.

In regard of radiotherapy education, there are three residence programmes in radiation oncology linked to universities at INEN, Social Security and Oncosalud and one Master Programme for Medical Physics.

Medical technologists have a five-year educational programme in private and public universities (including a one-year internship).

## ALATRO and the history of LATAM radiotherapy societies

The first radiotherapy society in LATAM was the *Círculo de Radioterapeutas Oncólogos Ibero-Latinoamericanos* (CRILA, Circle of Ibero-Latin American Radiation Oncologists) created under the initiative of Dr Raúl Vera in 1971 and integrated by 40 radiation oncologists from Argentina, Brazil, Chile, Colombia, El Salvador, Guatemala, México, United States and Venezuela. In subsequent years, oncologists from all LATAM countries joined CRILA. In 1991, the *Grupo Latino Americano de Curieterapia* (GLAC, Latin American Group of Curietherapy) was founded in the Gustave Roussy Institute, and was renamed in 1997 *GLAC Radio Oncológica* (GLAC-RO, Latin American Group of Radio-Oncological Curietherapy). In the year 2005, CRILA and GLAC-RO merged to form ALATRO, the Spanish acronym for *Asociación Ibero Latinoamericana de Terapia Radiante* [1]. ALATRO supports the development of radiotherapy in the region through: educational and research activities; sharing experiences; networking; advising on the development of policies and upholding quality measures for the protection of patients and radiation oncologists, physicists and medical technologists and harmonizing protocols.

## Radiation oncologists’ profile in Latin America

Radiation oncology in Latin America is developing but still faces problems and limitations. The specialty of radiation therapy is not homogeneous, and a few years ago, it was considered a subspecialty.

On the other hand, although the working conditions have improved for radiation oncologists, other professionals supporting radiotherapy activities face professional limitations. There are few medical physicists and their professional recognition is not adequate in most cases; there is a limited amount of continuing professional training and salaries are low (motivating multiemployment). Medical technologists face similar problems and they are in the process of recognition and replacing machine operators.

In an ALATRO workshop during this meeting the Montevideo Agreement (Acta de Montevideo) was approved in 2007. It states the following.

- The title of the specialty is defined by three terms: oncologist, clinician and radiotherapist.
- The radiation treatment of patients is a medical act indicated and performed under the direction of the clinical radio-oncologist, in which they participate with other physicians, physicists, technologists and nurses.
- If medications that modify the radiation effect are administered during radiation therapy, their use must be handled or coordinated by the radiation oncology clinician.
- For the next few years, radiation oncology clinicians will continue to be recognised as experts in the use of radiotherapy, including external radiotherapy, brachytherapy, radiosurgery and multimodal treatments for the cure of cancer and other diseases, as well as relieve patients’ symptoms.
- Through the relationship with their patients, the radiation oncology clinician will support and educate patients, helping them to understand their treatment options and navigate across the increasingly complex world of cancer treatment.
- Radiation oncology clinicians will increasingly consolidate their role within the multidisciplinary group and help clarify the nature of their specialty to their medical colleagues who refer their patients.
- Radiation oncology clinicians will increasingly be recognised for their unique perspective and understanding of the pathology, biology and clinical behaviour of cancer, their knowledge of physics and radiobiology and their expertise in the safe and accurate application of ionising radiation and radioactive isotopes.
- ALATRO will make every effort to ensure that its radiation oncology clinicians and professionals of related disciplines achieve these objectives through the following*.*
  1. Education: Providing professional development opportunities for all staff engaged in the management of cancer patients and in radiation treatment.
  2. Clinical excellence: Measuring and promoting the quality of care and evaluating its results.
  3. Research: Promoting this and applying its results in medical practice.
  4. Collaboration and communication: Improving these with colleagues, radiation oncology clinicians and other disciplines.
  5. Education to the public: Improving the diffusion of clinical radiation oncology and the use of ionising radiations.
- Those responsible for education in the specialty and dissemination of the discipline are universities, cancer institutes, specialised centres and authorised technical schools as well as national and international societies of the specialty. International agencies and organisations, such as the International Atomic Energy Agency and PAHO, are defined as collaborators for these purposes.
- The title must be the product of school-based training with university recognition, or of the academic institution or a recognised body that involves the following.
  1. Duration of three to four years.
  2. Graduated doctor.
  3. University training in institutes or centres adequately equipped.
  4. Inclusion of medical physics and radiobiology in the curriculum.
  5. Library facilities, Internet access and telemedicine.
  6. Facilities for research and publications.
  7. Additional training in internal medicine and/or surgery is recommended.
- The professional must have the skills to
  1. practice the discipline with competence and independence;
  2. integrate with multidisciplinary teams;
  3. exercise leadership in comprehensive cancer control and research programmes.
- Its general competences should be the following.
  1. To have theoretical and practical knowledge that allows the ethical, safe and efficient exercise of the specialty.
  2. Contribute to the development.
- As regards equipment, it is established that the immobilisation, simulation and computed planning systems should be generalised and that the use of cobalt equipment in external radiotherapy is absolutely legitimate.

### State of the art of radiotherapy in Latin America

There are great contrasts between LATAM countries, which have modern megapolises with 10–20 million inhabitants (Buenos Aires, Sao Paulo, Mexico City and Lima), while on the other hand, there are deserts and isolated cities. Large populated cities centralise healthcare facilities, including access to radiotherapy services. The equipment and implementation of radiotherapy facilities occurred in waves. In the 1950s, the equipment/inhabitants density was higher than the present day (due to the demographic effect), while cobalt-60 machines were implemented in Peru almost at the same time as in Spain.

An audit of the International Atomic Energy Agency (2008–2013) in 12 radiotherapy services located in ten LATAM countries (four in South America and six in Central America) identified several weaknesses regarding staff, infrastructure, processes and institutional organisation [22].

## Research and leading roles of LATAM radiation oncologists

Maybe the most prominent and vastly used contribution of Latin America to the radiotherapy field was the *Pasta Colombia*, developed by Dr Alfonso Eguerra-Gomez [5]. The *Pasta Colombia* consists of a mix of paraffin, beeswax and sawdust, which makes a flexible substance, mouldable at 45 °C (tolerable for patients) that allows the insertion of needles or tubes to facilitate contact brachytherapy.

Most of the published research in Latin America has been related to different strategies and results managing cervical and breast cancers in advanced stages, including irradiation of osteogenic sarcoma and nonconventional hypofractionation in advanced breast cancer [3, 6, 8, 13, 18, 19, 21, 23].

Several LATAM radiation oncologists had relevant political roles in their countries and in the region, and were involved in the development of cancer control plans such as Drs Mario Gaitán, Noriega Limón and Luis Pinillos Ashton. Also, opinions regarding international policies were published along with leading members of ALATRO [12, 14].

## Conclusion

It has been estimated that in developed countries, half of the new cases of cancer should receive radiotherapy at least once and up to 25% will receive a second course, while developing countries could have a greater need for radiotherapy because of their epidemiologic features and advanced disease burden [4].

We have presented some data about the initial steps of radiotherapy in Latin America, using the history of a few countries as an example, although this process was similar in other LATAM countries. Despite the fact that the current stage of development in radiotherapy varies between countries, they share common characteristics, for example, the presence of large urban areas offering state-of-the-art technology while other areas use simple techniques, and in some facilities, they work without physicists and in many areas they work without any radiotherapy facilities at all.

**Figure 5.**
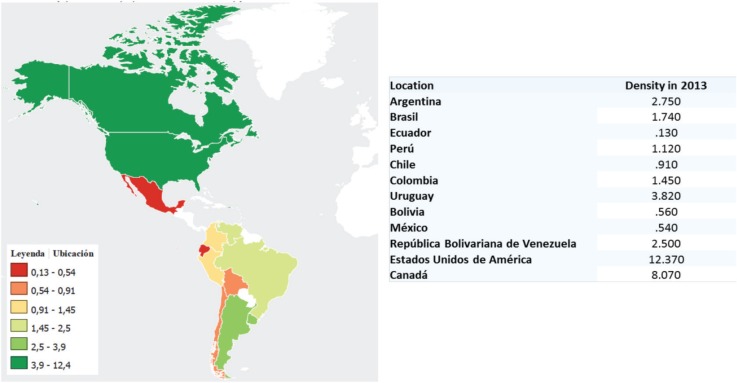
Density of radiotherapy units per million population in LATAM countries. Map of density elaborated in Knoema.com with data from UN World Health Statistics, 2014 (https://knoema.com/UNWHODATA2014/un-world-health-statistics-2014).

At the meeting of the Brazilian Society of Radiotherapy in 2016, the programme included a census where the representatives of different countries presented information regarding the equipment and facilities of their country (Table 1). Unfortunately, the results showed that the density of radiotherapy machines per population is much lower than developed countries such as Canada or the United States (Figure 5).

The profile for radiation oncologists, physicists and medical technologists has been well-defined, and further efforts should be made to comply with this standard. Universities and scientific societies should promote undergraduate and postgraduate training in basic and advanced concepts and certify in radiation oncology to guarantee quality and high standards.

Finally, national policies for cancer control plans should include the implementation of nationwide radiotherapy facilities to guarantee adequate access for cancer patients according to their needs.

## Figures and Tables

**Table 1.** Facilities of radiotherapy in selected LATAM countries^a^.

Countries/ Cancer incidence^b^ (new cases per year)	Services/ Centres	Megavoltage units/1000 cancer patients	External radiotherapy								Brachytherapy		
			Cobalt	Superficial RT	Accelerators	IMRT	IORT	SBRT	Radiosurgery	IGRT	HDR	MDR	LDR
Bolivia/11,286	8	1.33	5	3	3	—	—	—	—	—	1	—	6
Chile/40,415	23	1.26	2	—	40	—	—	—	2	3	7	—	2
Colombia/71,442	55	1.29	4	—	53	22	1	5	9	9	35	—	—
Costa rica/8948	4	1.01	3	1	5	—	1	—	—	—	1	—	—
Cuba/39,410	9	4.31	8	8	7	—	—	—	—	—	1	—	1
Ecuador/23,360	11	7.28	—	—	11	—	—	—	—	—	6	—	—
El salvador /3554	4	1.97	2	—	3	—	—	—	1	—	2	—	—
Guatemala/13,271	4	0.90	1	1	7	—	—	—	—	—	2	—	2
Honduras/7431	5	1.08	4	—	3	—	—	—	—	—	1	—	—
Mexico/65,540	23^c^	7.48	10	—	9	—	—	—	—	—	7	—	23
Nicaragua/5129	1	0.58	2	1	—	—	—	—	—	—	1	—	—
Panama/5415	4	1.48	—	—	6	—	—	—	—	—	1	—	1
Paraguay/8139	3	0.74	—	—	4	—	—	—	—	—	2	—	—
Peru/42,846	16	0.84	5	3	19	6	2	6	3	—	11	—	1
Puerto Rico/11,822	13	0.34	—	—	1	28	—	—	1	—	—	3	—
Dominican Republic/14,680	11	15.67	—	—	18	—	—	—	1	—	4	—	1
Uruguay/13,357	8	1.42	—	—	15	—	—	—	—	—	2	1	1

a data provided by ALATRO

b cancer incidence according to GLOBOCAN 2012

c data only for public centres
